# A Difference in Fatty Acid Composition of Isocaloric High-Fat Diets Alters Metabolic Flexibility in Male C57BL/6JOlaHsd Mice

**DOI:** 10.1371/journal.pone.0128515

**Published:** 2015-06-22

**Authors:** Loes P. M. Duivenvoorde, Evert M. van Schothorst, Hans M. Swarts, Ondrej Kuda, Esther Steenbergh, Sander Termeulen, Jan Kopecky, Jaap Keijer

**Affiliations:** 1 Human and Animal Physiology, Wageningen University, Wageningen, The Netherlands; 2 Department of Adipose Tissue Biology, Institute of Physiology of the Academy of Sciences of the Czech Republic v.v.i., Prague, Czech Republic; Faculty of Biology, SPAIN

## Abstract

Poly-unsaturated fatty acids (PUFAs) are considered to be healthier than saturated fatty acids (SFAs), but others postulate that especially the ratio of omega-6 to omega-3 PUFAs (n6/n3 ratio) determines health. Health can be determined with biomarkers, but functional health status is likely better reflected by challenge tests that assess metabolic flexibility. The aim of this study was to determine the effect of high-fat diets with different fatty acid compositions, but similar n6/n3 ratio, on metabolic flexibility. Therefore, adult male mice received isocaloric high-fat diets with either predominantly PUFAs (HFpu diet) or predominantly SFAs (HFs diet) but similar n6/n3 ratio for six months, during and after which several biomarkers for health were measured. Metabolic flexibility was assessed by the response to an oral glucose tolerance test, a fasting and re-feeding test and an oxygen restriction test (OxR; normobaric hypoxia). The latter two are non-invasive, indirect calorimetry-based tests that measure the adaptive capacity of the body as a whole. We found that the HFs diet, compared to the HFpu diet, increased mean adipocyte size, liver damage, and ectopic lipid storage in liver and muscle; although, we did not find differences in body weight, total adiposity, adipose tissue health, serum adipokines, whole body energy balance, or circadian rhythm between HFs and HFpu mice. HFs mice were, furthermore, less flexible in their response to both fasting- re-feeding and OxR, while glucose tolerance was indistinguishable. To conclude, the HFs versus the HFpu diet increased ectopic fat storage, liver damage, and mean adipocyte size and reduced metabolic flexibility in male mice. This study underscores the physiological relevance of indirect calorimetry-based challenge tests.

## Introduction

Excessive dietary fat intake is positively associated with weight gain and the development of metabolic diseases [1], such as insulin resistance, cardiovascular diseases, and type 2 diabetes. Metabolic diseases are often associated with a decrease in metabolic flexibility. Metabolic flexibility can be considered as the ability to switch between carbohydrate oxidation and fat oxidation [2] and is apparent in, for example, type 2 diabetics, who fail to adequately switch to glucose oxidation upon glucose consumption. Metabolic flexibility can, however, also be seen in a broader sense, in which it is defined as the capability to maintain homeostasis during a nutritional or environmental challenge [3]. Such a challenge can consist of administration of a single nutrient, such as glucose [4, 5] or lipid [6], or a combination of nutrients like in a meal test [7, 8]. Alternatively, it can consist of exposure to an environmental challenge, such as oxygen restriction (OxR), in which a major determinant for aerobic fuel oxidation—oxygen—is limited [9, 10]. An adequate response to the challenge, ultimately, requires substrate switching, which is impaired when metabolic flexibility declines. Metabolic inflexibility arises from deficiencies in the handling of incoming and circulating nutrients by one or several of the organs that play a major role in metabolism, such as the liver, skeletal muscle, adipose tissue, brain or pancreas [2]. Detection of metabolic inflexibility would, thus, benefit from a multiple level approach, in which different organs or metabolic systems are challenged.

Next to the amount of dietary fat, the composition and type of fat affect the development of metabolic disease and metabolic inflexibility. Saturated fatty acids (SFAs) increase fasting insulin levels, weight gain, circulating leptin levels, liver triacylglycerols, mean adipocyte size and adipose tissue inflammation, and decrease adiponectin levels, compared to poly-unsaturated fatty acids (PUFAs) [11–18]. Furthermore, the dietary proportion of fatty acids to each other seems to determine health effects [13]. Among the SFAs, for example, medium chain SFAs protect against the detrimental effects of long chain SFAs in both humans and rodents [19, 20]; and among the PUFAs, omega-3 PUFAs (n-3 PUFAs) were shown to be more beneficial for rodent health status than omega-6 PUFAs (n-6 PUFAs) [21–23] that appear to have pro-inflammatory properties [24]. It has, indeed, been suggested that especially the ratio of n-6 PUFAs to n-3 PUFAs (n6/n3 ratio) determines the pathogenesis of metabolic diseases, rather than the absolute amounts of n-3 PUFAs and n-6 PUFAs [25]. The influence of the dietary fatty acid composition on metabolic flexibility, however, remains largely unknown. In this study we, therefore, investigated the effects of two isocaloric high-fat (HF) diets that differ in fatty acid composition on metabolic health and metabolic flexibility in mice.

The first diet (HFpu diet) is a standardized HF diet that was used in several previous studies [9, 26–28] and mainly contains PUFAs in the fat fraction. The second diet (HFs diet) is identical to the HFpu diet, except for the fat fraction that mainly contains saturated fatty acids from palm oil. Palm oil is the dominant fat constituent of most experimental rodent HF diets and is increasingly prevalent in human food products. Although the HFpu diet contains much more PUFAs, the ratio of n-6 FAs to n-3 FAs was kept similar between both diets.

Metabolic flexibility was measured with one invasive and two non-invasive challenge tests. The oral glucose tolerance test (OGTT) is an invasive challenge test that monitors the homeostatic blood glucose clearance in response to a glucose challenge. The two non-invasive challenges were monitored with indirect calorimetry instead of blood sampling. Indirect calorimetry-based challenge tests are suitable to assess metabolic flexibility because indirect calorimetry directly displays the extent and time course of substrate switching. For this study, we used indirect calorimetry to monitor the response to a nutritional challenge, a fasting and re-feeding challenge, and to an environmental challenge, a mild reduction in the availability of oxygen (OxR: [O_2_] = 12% vs. 21% in normoxia). Using food as a challenge will target a wider range of processes than during an OGTT and, therefore, provides a broader reflection of metabolic sensitivity. OxR forces the use of metabolic pathways that facilitate adaptation to decreased oxygen availability and thus directly targets flexibility. Body weight, adiposity, liver and adipose tissue health, and circulating hormone and adipokine levels were also monitored during the course of the experiment to evaluate metabolic health status.

## Materials and Methods

### Animals and experimental manipulations

Thirty male C57BL/6JOlaHsd mice were used for this study (Harlan Laboratories, Horst, The Netherlands). The experimental protocol was approved by the Animal Welfare Committee of Wageningen University, Wageningen, The Netherlands (DEC2012088). Mice arrived at 10 weeks of age and were individually housed and maintained under environmentally controlled conditions (21 ± 1°C, 12 h/12 h light—dark cycle, 50 ± 10% relative humidity) and had *ad-libitum* access to feed and water. The study consisted of a three week adaptation phase and an experimental phase of 27 weeks (wk1–wk27). During the adaptation phase, mice received the purified low-fat BIOCLAIMS standard diet, which contains 10% energy from fat [26]. For the experimental phase, mice were stratified by body weight and allocated to the HFpu or HFs group. Both high-fat diets contained 40% energy from fat, but differed in the composition of the fat component. The control diet is the BIOCLAIMS purified standardized HF diet (HFpu diet), with 70% (w/w) sunflower oil, 18% (w/w) coconut oil and 12% (w/w) flax seed oil [27]. The HFs diet contains 98.1% (w/w) palm oil and 1.9% (w/w) flax seed oil (for a detailed description of both diets, see Table 1). The HFs diet thus contained more saturated and long chain fatty acids, and less medium chain and n-3 fatty acids than the HFpu diet. The HFs contained the minimal requirements for n-3 fatty acids [29] and the ratio of n6/n3 fatty acids was kept similar between both diets.

**Table 1:** Diet composition of HFpu and HFs.

Ingredients (g kg^-1^)	HFpu	HFs
Casein	267	267
Wheat starch	172.5	172.5
Maltodextrin	100	100
Glucose	50	50
Sucrose	100	100
Coconut oil	37.8	0
Sunflower oil	147	0
Flaxseed	25.2	4
Palm oil	0	206
Cholesterol	97 mg	97 mg
Cellulose	50	50
Mineral mixture	35	35
Vitamin mixture	10	10
Choline bitartrate	2.5	2.5
L-Cysteine	3	3
Energy (kJ g^-1^)	19.7	19.7
Energy (% of total energy content)		
Carbohydrate	36.8	36.8
Fat	40.2	40.2
Protein	23.0	23.0
Fatty acid profile (g kg^-1^)		
Caprylic acid (C8:0)	2.8	0
Capric acid (C10:0)	2.3	0
Lauric acid (C12:0)	16.9	0.2
Myristic acid (C14:0)	6.4	2.1
Palmitic acid (C16:0)	13.1	89.8
Stearic acid (C18:0)	8.5	9.0
Oleic acid (C18:1 n-9)	35.5	76.1
Linoleic acid (C18:2 n-6)	100.9	19.3
α-Linolenic acid (C18:3 n-3)	13.5	2.5
Fatty acid profile (% of total energy content)		
Saturated fatty acids	9.6	19.4
Medium chain fatty acids (C6:0 –C12:0)	4.2	0
Long chain fatty acids (C14:0- C18:0)	5.4	19.3
Mono-unsaturated fatty acids	6.8	14.6
Poly-unsaturated fatty acids	21.9	4.2
Omega 3 fatty acids	2.6	0.5
Omega 6 fatty acids	19.3	3.7
Ratio n-6/n-3 fatty acids	7.5	7.6

Body weight, body composition, by using an EchoMRI Whole Body Composition Analyser (EchoMRI, Houston, USA), and feed intake were monitored on a weekly basis. Mice received a fresh batch of feed at the start of each week and feed intake was determined from week 5 onwards by weighing the amount of feed at the start and at the end of each week. Three mice of each dietary group were sacrificed after 5 days of HF-feeding to analyse liver steatosis at the start of the experiment. The remaining 12 mice per dietary group continued until the end of the experiment; although, 1 mouse of the HFpu group died before the end of the experiment for reasons unrelated to the dietary intervention and was excluded from all analyses. Indirect calorimetry was performed in weeks 5 and 20. OGTTs were performed in week 22; the response to fasting and re-feeding and the response to OxR was measured in week 25. Mice were sacrificed in week 27 by decapitation—to prevent effects of anaesthesia on metabolic parameters [30, 31], after which blood was collected in Mini collect serum tubes (Greiner Bio-one, Longwood, USA), and centrifuged for 10 minutes at 3000 g and 4°C to obtain serum. Serum samples were aliquoted and stored at -80°C. Glucose concentration was measured in whole blood with a Freestyle blood glucose system (Abbott Diabetes Care, Hoofddorp, the Netherlands) according to the manufacturer’s instructions. After blood collection, liver tissue, the left epididymal WAT (eWAT) depot and the acromion trapezius muscle, which is situated between the shoulder blades and underneath the brown adipose depot, were dissected and snap frozen in liquid nitrogen and stored at −80°C. The right eWAT depot was weighted and stored in Dulbecco’s Phosphate Buffered Saline (PBS; Gibco, Paisley, UK) with 3.7% (v/v) formaldehyde (Merck KGaA Darmstadt, Germany) (pH = 7.40) at 4°C.

The macronutrient content of the HFs diet was matched to the macronutrient content of the HFpu diet, which is a standardized high-fat diet that we also used in previous studies [26]. Diets only differ in the amount and type of dietary oil that was added to obtain two isocaloric diets with 40% energy from fat. The fatty acid profile of the used dietary oils was derived from the USDA National Nutrient Database for Standard Reference [32].

### WAT and liver histology and mitochondrial density

The caudal part (one third of the complete tissue) of the right eWAT depot was fixed in PBS with 3.7% (v/v) formaldehyde (pH = 7.40) at 4°C for 24 hours with moderate shaking, and then washed in PBS for 2 hours with moderate shaking and stored in PBS with 0.1% (w/v) sodium azide for several days at 4°C until further use. Fixed tissues were embedded in paraffin and sectioned at 5 μm. Adipocyte size and the amount of crown-like structures (CLS) in eWAT were determined as published [33] with adaptations as described [28, 34] in 8 mice per dietary group (randomly selected) and used as an indication of adipose tissue health [35]. Briefly, macrophages were stained with a monoclonal anti-MAC2 antibody (diluted 1:5000, overnight incubation at 4°C; Cedarlane Laboratories Limited, Burlington, Canada). Next, sections were rinsed in PBS and incubated for 60 min at room temperature with a secondary goat anti-rat biotinylated antibody (diluted 1:200; Vector laboratories, Burlingame, CA, USA), rinsed again with PBS, and incubated for 60 min at room temperatures with Vectastain Elite ABC reagent (dilution 1:1000; Vector laboratories). After rinsing with PBS, the sections were incubated for 2 minutes at room temperature with 3,3′-diaminobenzidine solution (dilution 1:200; Vector laboratories). To determine adipocyte size, sections were stained with haematoxylin QS (Vector laboratories). To determine adipocyte size, we used sections from three distant layers of the adipose depot (with 150 μm distance between layers). Within each layer, approximately 5 pictures were taken with a Zeiss Axioscope 2 microscope equipped with an axioCamMRc 5 digital camera (Carl Zeiss, Jena, Germany). All adipocytes in each picture were manually encircled with the Axiovision software (Carl Zeiss, version 4.8) to determine the surface area of at least 400 adipocytes per mouse. The number of CLS was scored manually in 1000 adipocytes per mouse in the same areas as the sections that were used to determine adipocyte size. All analyses were performed blinded to dietary background.

Mitochondrial density in eWAT was determined as indicator of WAT health. The ratio of mitochondrial DNA to nuclear DNA was measured with reverse transcription quantitative real-time PCR (RT-qPCR) as published [36] and with modifications as described [10]. Briefly, total DNA was extracted from homogenized eWAT by digestion with Proteinase K (Sigma-Aldrich, St Louis, USA) in a lysis buffer (50 mM Tris-HCL, pH 7.5, 0.5% (w/v) SDS and 12.5 mM EDTA, pH 8.0) and RNAse A (Sigma-Aldrich). Samples were then centrifuged, after which the aqueous phase was mixed and extracted with phenol-chloroform-isoamylalcohol and twice with chloroform. DNA was precipitated by 96% (v/v) ethanol and sodium acetate (3M, pH 5.2), washed with cold 70% (v/v) ethanol, air-dried and re-suspended in 10 μl of RNAse DNAse free water. The quality and quantity of DNA in each sample were analysed with the Nanodrop (IsoGen Life Science, Maarssen, The Netherlands) and each sample was diluted to the same DNA concentration of 100 ng μL^-1^.

### Serum and tissue analyses

Serum insulin, leptin and adiponectin levels were determined with the Bio-Plex Pro Mouse Diabetes Assay (Bio-Rad, Veenendaal, The Netherlands) according to the instructions of the manufacturer with the Bio-Plex 200 system (Bio-Rad) and used as indicators of adipose tissue health. Serum aspartate transaminase and alanine transaminase levels were determined with the AST and ALT enzymatic assay kit (Bioo Scientific Corporation, Austin, USA) according to the instructions of the manufacturer. Triacylglycerol levels in liver and in the acromion trapezius muscle were determined with the Triglycerides Liquicolor Kit (Human, Wiesbaden, Germany) according to the instructions of the manufacturer and as published [37]. Approximately 20 mg of liver or muscle tissue was grinded to a powder in liquid nitrogen. Grinded tissue was dissolved in homogenisation buffer (10 mM TRIS-HCl, 2mM EDTA, 250 mM sucrose, pH 7.5) to a concentration of 40 mg tissue/ ml buffer. Tissue homogenates were homogenized with an automated pellet mixer (VWR, Boxmeer, The Netherlands) for at least one minute and by pulling the homogenate through a 25 gauge needle at least three times until all tissue was fully dissolved.

The fat fraction of the liver (n = 7–8) and the diets (in triplicate) was extracted using the Bligh-Dyer protocol [38]. The concentration and partial composition of phospholipids and triacylglycerols in liver tissue and in the diets were determined with shotgun lipidomics as published [39]. Before the analysis, diets were stored for a week at 22°C to measure potential dietary fatty acid oxidation, which was not observed (data not shown).

### Oral glucose tolerance test (OGTT)

On the day of the OGTT, food was removed 1.5 hour after start of the light phase. Mice remained without food for the following 5 hours, after which blood glucose was measured via a tail cut with the Freestyle blood glucose system (Abbott Diabetes Care) and 2 g glucose /kg bodyweight was given by oral gavage. Fifteen, 30, 60, 90, and 120 min after glucose administration, glucose concentration was determined using the Freestyle blood glucose system. Glucose tolerance was analysed with individual time course data of blood glucose levels and the individual incremental area under the curve (iAUC).

### Indirect calorimetry

Oxygen consumption and carbon dioxide production were measured with an open indirect calorimetry system (TSE Systems, Bad Homburg, Germany) that is equipped for simultaneous measurements of 12 individual mice, as published [40]. Mice could adapt to the indirect calorimetry system for 24 hours, after which the actual measurement started and O_2_ consumption and CO_2_ production were recorded every thirteen minutes. Energy expenditure (EE), respiratory exchange ratio (RER) and physical activity were determined as described previously [10].

To measure the response to fasting and re-feeding, mice received 1.5 gram of their own feed at the start of the dark phase (18.00h) in the indirect calorimetry system. All feed was consumed within the first 6 hours of the dark phase, after which the RER started to decline. Between 6.00h and 14.00h of the following light phase, mice were in a fasted state (RER<0.75). At 14.00h, mice regained *ad-libitum* access to the HFpu diet (HFpu mice) or HFs diet (HFs mice). RER values of each mouse were averaged during the period of fasting (from 23.00h to 6.00h), during the period when mice are fasted (from 6.00h to 14.00h) and during the period of re-feeding (from 14.00h to 22.00h). The maximal RER-value during re-feeding was calculated as the average of the three highest consecutive RER values representing a time span of 26 minutes during the complete period of re-feeding and was used for comparison with the food quotient of both diets. Food quotients were calculated with the heat equivalents of CO_2_ of each component of the diet [41]. Heat equivalents of the individual dietary oils were derived from Livesey and Elia [42]. The percentage of energy from glucose oxidation was derived from average RER values of individual mice with the table of Peronnet [43].

Indirect calorimetry during OxR was performed as described [9, 10]. For the exposure to OxR we used the same fasting regime as during the response to fasting and re-feeding (mice received 1.5 gram of feed at the start of the dark phase). One hour after start of the following light phase, oxygen concentration in each animal cage was decreased to 12% and O_2_ consumption and CO_2_ production were recorded every thirteen minutes for the following 6 hours. The day previous to the exposure to OxR, mice were kept in the indirect calorimetry system under the same (fasting) conditions and during the same time period of the day but under normoxic conditions, which means that in the analysis to the response to OxR, mice served as their own control. Blood glucose was measured directly after the exposure to OxR or normal air via a tail cut and with the Freestyle blood glucose system (Abbott Diabetes Care).

### Statistics

Data are expressed as mean ± SEM. All analyses are based on the data of the 11 HFpu mice and 12 HFs mice; except for the analysis of adipocyte size and WAT CLS, which is based on 8 mice per group. Statistical analyses were performed using GraphPad Prism version 5.04 (Graphpad, San Diego, USA). Data were checked for normality using the D’Agostino and Pearson omnibus normality test. Test results that were not normally distributed were log-transformed or analysed with a non-parametric test (feed intake in the indirect calorimetry system wk20). Measurements at single time points between 2 independent groups were analysed by independent Students’ *t*-tests.

Comparisons between two groups that were repeated over time were analysed with two-way repeated measures ANOVA (factor 1 = diet, factor 2 = time) and Bonferroni post-hoc analysis. *P*-values smaller than 0.05 were considered to be statistically significant.

## Results

### The HFs diet mainly affected mean adipocyte size in eWAT and ectopic lipid storage in liver and skeletal muscle

**Fig 1:**
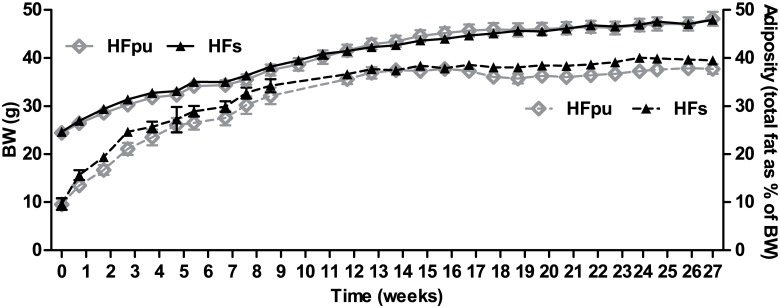
Body weight and total adiposity during 27 weeks of HF feeding. Body weight (solid lines) and total adiposity (dotted lines) were determined on a weekly basis during the 27 weeks of HFpu and HFs feeding. Total adiposity is expressed as the percentage of total body fat over body weight.

After 27 weeks of HF feeding, we detected no differences in BW, adiposity (Fig 1), cumulative feed intake, WAT mitochondrial density, serum glucose, insulin, leptin, or adiponectin levels (Table 2) between HFpu and HFs mice. Of note, both groups of mice had relatively high fasting insulin levels that are well above levels that are usually seen in high-fat fed male C57BL/6J mice (around 0.11 nM after 18 weeks of feeding [44]).

**Table 2:** Phenotypical data of mice fed the HFpu or HFs diet for 27 weeks.

	HFpu	HFs	*p*-value
Cumulative feed intake (kg)	0.350 ± 0.007	0.339 ± 0.005	n.s.
Serum insulin (nM)	2.36 ± 0.17	1.92 ± 0.20	n.s.
Serum glucose (mM)	6.90 ± 0.19	7.16 ± 0.15	n.s.
Serum leptin (nM)	6.13 ± 0.71	5.67 ± 0.94	n.s.
Serum adiponectin (mg L-^1^)	15.5 ± 1.10	18.9 ± 1.35	n.s.
Serum triacylglycerols (g L^-1^)	6.02 ± 0.29	6.69 ± 0.57	n.s.
WAT mitochondrial density (ratio to HFpu)	1.00 ± 0.09	0.87 ± 0.07	n.s.
eWAT weight (g)	0.795 ± 0.067	0.866 ± 0.036	n.s
Liver triacylglycerol (mg g^-1^ liver)	52.7 ± 3.30	66.1 ± 3.02	0.007
Muscle triacylglycerols (mg g^-1^ muscle)	17.9 ± 1.61	22.9 ± 1.67	0.045
Serum aspartate transaminase activity (μM s^-1^)	0.69 ± 0.06	1.07 ± 0.12	0.009
Serum alanine transaminase activity (μM s^-1^)	0.83 ± 0.06	1.51 ± 0.16	<0.001

Data are expressed as mean ± SEM (n = 11 for HFpu and n = 12 for HFs). All parameters were measured after 27 weeks of HFpu or HFs feeding and when mice were in a fasted state. The cumulative feed intake is calculated from the total feed intake between week 4 and week 27. eWAT weight represents the weight of the right epididymal WAT depot only. n.s. non-significant.

**Fig 2:**
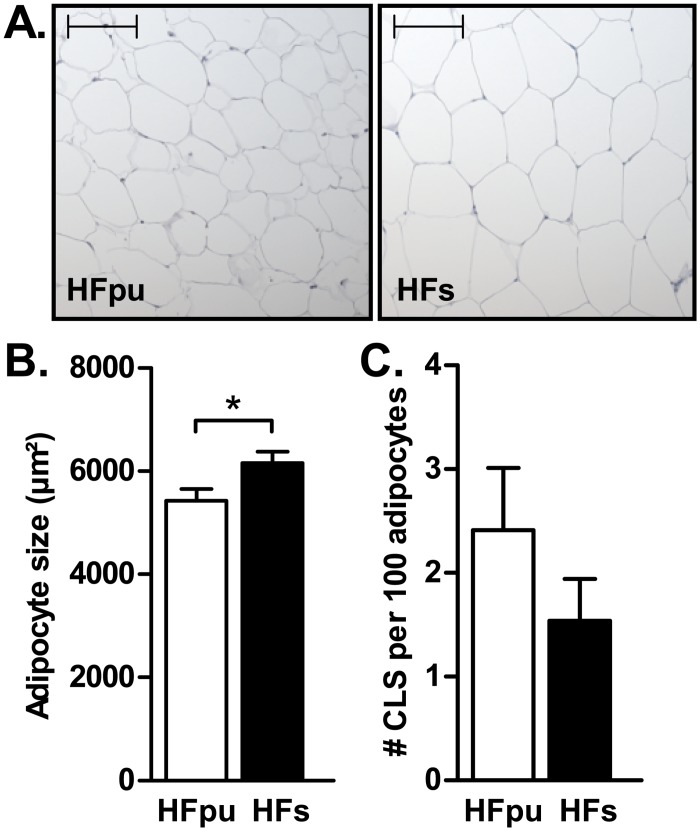
Mean adipocyte size and prevalence of crown-like structures in eWAT after 27 weeks of HF feeding. Representative images (A) of the haematoxylin stainings that were used to determine the average adipocyte surface area (B) in eWAT of HFpu and HFs mice. The bar in each picture represents a distance of 100 μm. The number of CLS (C) was determined with a MAC-2 macrophage staining in eWAT. * P< 0.05 HFs mice vs. HFpu mice.

Adipocytes in eWAT were significantly larger in HFs mice compared to HFpu mice (Fig 2A and 2B), whereas no difference in the number of crown-like structures in eWAT was observed (Fig 2C). HFs mice had significantly higher triacylglycerol levels in liver and muscle (Table 2), which indicates increased ectopic lipid storage. Consistently, serum aspartate transaminase and alanine transaminase levels, markers for liver damage, were both significantly increased in HFs mice compared with HFpu mice (Table 2). To obtain an impression of the extent of liver steatosis in week 27 compared to the situation at the start of the intervention, hepatic lipids were stained with Oil-red-O after either 5 days or 27 weeks of high-fat feeding in a limited number of mice per group (S1 Fig). Both the number and size of hepatic lipid droplets strongly increased over time.

Lipidomic analysis revealed that the hepatic phospholipid composition was not affected by the lipid composition of the diet (data not shown). The liver triacylglycerol fraction mirrors the lipid composition of the diet with respect to the distribution of the number of double bonds (S2A Fig), which reflects the degree of unsaturation). Indeed, significant differences were observed when the major (TAGs C50-C54) triacylglycerols were analysed (S2B Fig). Differences in liver triacylglycerol fatty acid chain length were not observed (S2C Fig).

### Substrate oxidation and energy expenditure under non-challenged conditions do not explain the increase in adipocyte size and ectopic lipid storage

Energy expenditure, physical activity and the respiratory exchange ratio under non-challenged conditions were measured with indirect calorimetry in week 5 and week 20 of HF feeding (Table 3). We also analysed diurnal (or circadian) rhythm, since it is known that a disturbance in circadian rhythm relates to metabolic dysfunction [45]. To ensure accurate measurement of EE, RER and physical activity, we also analysed 24h feed and water intake in the indirect calorimetry system and compared that with the feed intake in the home cage and between dietary groups. A drop in feed intake might indicate stress and disturbs the measurement of diurnal RER. EE and physical activity can also be affected by stress.

**Table 3:** Indirect calorimetry measurements of HFpu and HFs mice in week 5 and week 20.

	Week 5		Week 20		
	HFpu	HFs	HFpu	HFs	sign.
Respiratory exchange ratio	0.832 ± 0.008	0.812 ± 0.011	0.836 ± 0.007	0.829 ± 0.008	n.s.
Energy expenditure (J s^-1^)	0.583 ± 0.008	0.575 ± 0.009	0.684 ± 0.018	0.678 ± 0.013	§§§§
Activity (total beam breaks (x1000))	36.4 ± 2.94	26.5 ± 2.03	25.8 ± 2.89	19.7 ± 1.37	§§§, ŦŦ
% of activity in the DP	70.0 ± 3.46	67.1 ± 1.57	64.9 ± 3.57	66.5 ± 1.44	n.s.
Feed intake (g)	2.77 ± 0.10	2.61 ± 0.24	3.14 ± 0.13	2.95 ± 0.22	n.s.
% of FI in the DP	70.4 ± 2.12	66.1 ± 5.74	63.8 ± 3.30	68.5 ± 2.94	n.s.
Water intake (ml)	1.74 ± 0.16	1.45 ± 0.20	2.25 ± 0.21	1.84 ± 0.14	§§

Data are expressed as mean ± SEM (n = 11 for HFpu and n = 12 for HFs). Indirect calorimetry measurements during normal, free-feeding conditions were performed after 5 and after 20 weeks of feeding the HFpu or HFs diet. Data were recorded and averaged over 24 hours. Feed intake (FI) and physical activity are also expressed as the percentage in the dark phase (DP) to give insight into the diurnal pattern.

§ indicates a significant effect of time (§§ *P*<0.01, §§§ *P*<0.001 and §§§§ *P*<0.0001).

Ŧ indicates a significant effect of the diet (ŦŦ *P*<0.01).

n.s. non-significant.

Diurnal feed and water intake in the indirect calorimetry system were not significantly different between both dietary groups (Table 3). Feed intake in the indirect calorimetry system was, furthermore, not significantly different from feed intake in the home cage. Feed intake (g) in the home cage was 2.98 ± 0.05 for HFpu and 2.88 ± 0.05 for HFs in week 5 and 3.39 ± 0.08 for HFpu and 3.28 ± 0.05 for HFs in week 20.

HFpu mice had significantly higher physical activity levels compared with HFs mice. The increase in physical activity did, however, not result in a significant increase in EE. HFpu and HFs mice did not differ in average diurnal RER levels. Physical activity was significantly higher in week 5 compared to week 20 for both groups of mice, whereas energy expenditure was higher in week 20 compared to week 5.

Finally, we analysed the diurnal pattern with the percentage of physical activity and the percentage of feed intake in the dark phase. Both HFpu and HFs mice were more active and consumed more feed during the dark phase in both measurements (p<0.0001 for all 4 comparisons) as expected for nocturnal animals. HFpu and HFs mice did not differ in the percentage of physical activity or the percentage of feed intake in the dark phase, which shows that they have similar diurnal patterns.

### Assessment of metabolic flexibility

**Fig 3:**
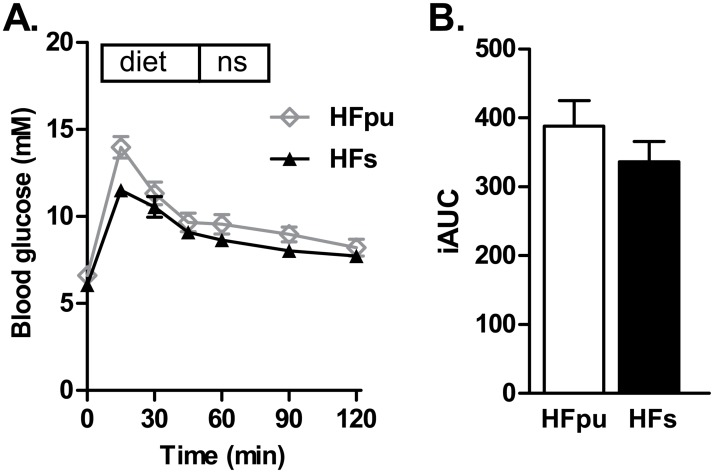
Oral glucose tolerance test after 22 weeks of HF feeding. HFpu and HFs mice were fasted for 5 hours at the start of the light phase, after which mice received glucose via oral gavage. Blood glucose levels were measured before glucose administration and during the 2 hours thereafter (A), and were expressed as iAUC (B).

One of the major aims of this study was to investigate functional implications of changes in health status by assessing metabolic flexibility. The first test that we used was a standardized OGTT. The two isocaloric HF diets showed a comparable glucose tolerance after 22 weeks of feeding (Fig 3A and 3B).

**Fig 4:**
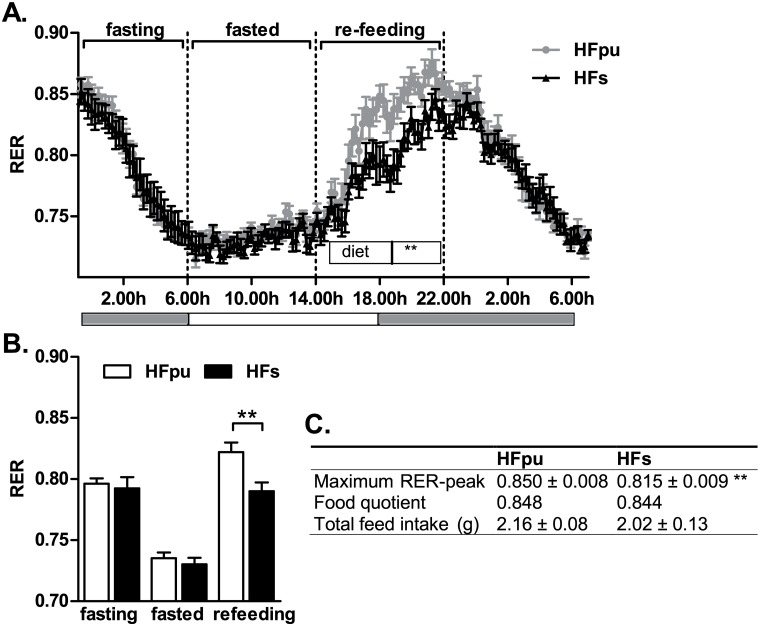
RER during the fasting and re-feeding challenge after 25 weeks of HF feeding. HFpu and HFs mice were fasted and regained *ad-libitum* access to their feed at 14.00h, while RER was monitored continuously (A, the grey bars below the figure indicate the dark phase). RER values of individual mice were averaged when mice were fasting (from 23.00h to 6.00h), when mice were in a fasted state (from 6.00h to 14.00h) and during re-feeding (from 14.00h to 22.00h) (B). The table (C) summarises the maximal RER-value during re-feeding based on three consecutive measures, the food quotients of the diets, and the feed intake during the 8 hours of re-feeding. ** *P*< 0.01 HFs mice vs. HFpu mice.

Metabolic flexibility was also assessed with a fasting and re-feeding test. HFpu and HFs mice had low RER values when fasted, which indicates preferential whole body fat oxidation. During re-feeding, RER increased, which indicates a combination of both fat and carbohydrate oxidation on whole body level (Fig 4A). HFpu and HFs mice did not differ in mean RER during fasting (from 23.00h to 6.00h) or when they were in a fasted state (from 6.00h to 14.00h). During re-feeding (from 14.00h to 22.00h), however, HFpu mice showed a significantly larger increase in RER compared to HFs mice. Furthermore, the average RER over the complete period of re-feeding (Fig 4B) and maximal RER-value during re-feeding (Fig 4C) of HFpu mice matched the food quotient of the HFpu diet, which indicates that HFpu mice effectively alter their metabolism to use the nutrients that are available in the diet. In contrast, HFs mice did not increase RER levels above 0.815, which is significantly lower than the levels that were obtained in HFpu mice and is remarkably lower than the food quotient of the HFs diet. During re-feeding, mice had *ad-libitum* access to feed, during which HFpu and HFs mice consumed a similar amount of feed (Fig 4C).

**Fig 5:**
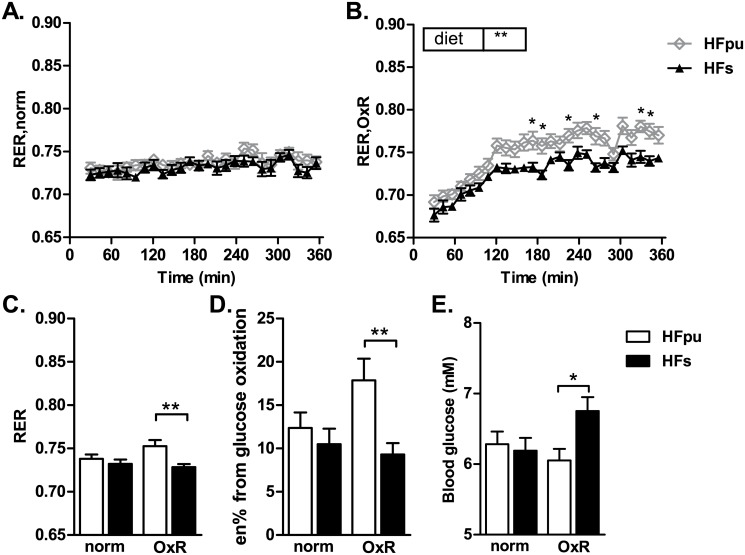
RER during the OxR challenge after 25 weeks of HF feeding. HFpu and HFs mice were fasted and exposed to normoxic air (A; 20.8% O_2_) or to oxygen restriction (B; 12.0% O_2_) for 6 hours. Asterisks (in B) indicate the individual time points at which the 2-way ANOVA post-hoc analysis revealed significant differences between HFpu and HFs mice. RER values of individual mice were averaged during the 6 hours in normal or hypoxic air (C) and used to estimate the percentage of energy (en%) from glucose oxidation (D). Blood glucose was measured directly after the exposure to OxR or normal air (E). * *P*<0.05 and ** *P*< 0.01 HFs mice vs. HFpu mice.

Finally, metabolic flexibility was assessed using an exposure to OxR. HFpu and HFs mice did not differ in RER under normoxic conditions (Fig 5A). Exposure to OxR increased RER in HFpu mice compared to HFs mice (Fig 5B and 5C), which translates to an increase in the percentage of energy from glucose oxidation (Fig 5D). An increase in glucose oxidation is considered advantageous during OxR since the oxidation of glucose requires less oxygen than the oxidation of an equimolar amount of fat. Furthermore, an increase in glucose oxidation can prevent the increase in blood glucose levels that is often seen during OxR [9]. The analysis of blood glucose levels before and after exposure to OxR revealed a (statistical) interaction between the diet (HFpu and HFs) and oxygen level (OxR and normoxia) because HFpu and HFs responded differently to the exposure. Bonferroni post-hoc analysis revealed that HFs mice had higher blood glucose levels during OxR compared to HFpu mice (Fig 5E).

## Discussion

We examined the effects of two isocaloric high-fat diets differing in fatty acid composition, but similar n6/n3 ratio, on metabolic flexibility and different markers of metabolic health in adult male mice. The HFpu diet contained predominantly PUFAs, while the HFs diet mainly contained SFAs, supposedly being unhealthier. Unexpectedly, most biomarkers, including body weight, adiposity, WAT health, serum glucose, insulin and adipokine levels, and glucose tolerance did not differ between the two dietary groups. HFs mice, however, had bigger adipocytes, higher triacylglycerol levels in liver and skeletal muscle, higher serum alanine transaminase and aspartate transaminase levels, and a reduced physical activity level. Despite these relatively small phenotypical changes, HFs mice were less flexible in the response to the fasting and re-feeding challenge and the OxR challenge, which indicates a reduction in metabolic flexibility.

### The ratio of n-6 to n-3 PUFAs might account for the small phenotypical differences between HFpu and HFs mice

The HFpu and HFs diets primarily differ in the amount of long chain SFAs (5.4% in HFpu vs. 19.3% in HFs) and in the amount of PUFAs (21.9% in HFpu vs. 4.2% in HFs). Excessive consumption of saturated fat leads to WAT expansion, WAT inflammation and inhibition of glucose and fat oxidation (reviewed by [17]). Long chain SFAs (C14-C18), compared to medium chain SFAs (C6–C12), further increase weight gain and fat-mass gain [13, 46]. n-3 PUFAs, on the other hand, reduce weight gain [47], protect against cardiovascular diseases [48] and reduce inflammatory proteins [49]. These effects seem to contrast our results. However, differences in weight gain, WAT inflammation, or adiposity are not always reported in studies in which the amount of SFAs is increased compared to other (unsaturated) fatty acids [12–14, 22]. Moreover, studies in which the amount of n-3 PUFAs is increased compared to other (saturated or n-6) FAs do not always show improvements in body weight, liver lipids or inflammatory cytokines [50–53].

One explanation for the absence of major differences in the studied markers for metabolic health between HFpu and HFs mice may be that both diets have a similar n6/n3 PUFA ratio. It has been proposed that health effects of HF diets primarily depend on this ratio [25]. A ten-fold increase in the n6/n3 ratio in isocaloric high-fat diets with similar SFA content, for example, increased hepatic lipid storage, even though feed intake was significantly higher in the low n6/n3 group [54]. It was also shown that a 4-fold increase in the amount of SFAs did not increase body weight, WAT inflammation or adipocyte size when the n6/n3 ratio was kept equal between diets [13]. However, there are also studies that show that the n6/n3 ratio does not influence body weight [55] or lipid peroxidation in liver [56], suggesting that the absolute mass of essential fatty acids, rather than the n6/n3 ratio, determines long-term health effects of a diet [57]. Our study, however, shows that body weight gain and WAT health are similarly affected by diets differing in fatty acid composition, but with similar n6/n3 ratio.

### The HFs diet and HFpu diet differently affect lipid storage in WAT, muscle and liver

After 27 weeks of high-fat feeding, HFs mice had bigger adipocytes and more ectopic lipid storage in muscle and liver than HFpu mice, which suggests that the type of dietary fat affects the allocation of fat storage. Adipocyte hypertrophy is known to indicate a reduced capacity to initiate hyperplasia [58]. A certain reduction in the formation of new adipocytes increases fat storage in existing adipocytes, leading to further adipocyte enlargement. Large adipocytes are less sensitive to insulin than smaller adipocytes (reviewed by [59]) and adipocyte size is positively associated with insulin resistance and Type 2 diabetes in both humans and rodents [60, 61]. The failure to recruit new adipocytes, furthermore, does not only increase adipocyte hypertrophy but also increases ectopic fat storage [58]. Ectopic fat deposition, or steatosis, can lead to cell dysfunction and cell death [62] and is strongly associated with insulin resistance [63]. n-3 PUFAs are known to prevent both adipocyte hypertrophy and obesity-related adipose remodelling [64], which might explain the increase in adipocyte size, and possibly related ectopic lipid storage, in HFs vs. HFpu mice.

The increase in ectopic lipid accumulation in HFs mice might, on the other hand, also result from increased ectopic lipid uptake, increased fatty acid synthesis, or reduced fat oxidation in liver and muscle tissue [65], which are known to be affected by dietary fat composition [14, 66, 67]. The triacylglycerol composition in the liver suggests that dietary fatty acids are remodelled by especially elongases to meet specific metabolic requirements; the pattern of fatty acid unsaturation of the diet is, however, still observable.

The dietary fatty acid composition, thus, seems to alter the allocation of fat storage, even though total adiposity remains equal between both groups. The increase in ectopic lipid storage is expected to lead to differences in metabolic health and metabolic flexibility.

### The changes in the response to fasting and re-feeding and oxygen restriction in HFs mice indicate impairment of carbohydrate metabolism

Despite the significant changes in adipocyte size and ectopic lipid storage, HFs and HFpu mice did not differ in in their response to the OGTT. HFs and HFpu mice may have a similar glucose tolerance because both diets provide a similar glycaemic load. Next to the OGTT, metabolic flexibility was analysed with two non-invasive tests in the indirect calorimetry system. Under unchallenged conditions, HFpu and HFs mice did not differ in mean diurnal RER; furthermore, diurnal RER values matched the food quotient of the diet consumed, which means that under free-feeding conditions, macronutrient oxidation matches macronutrient availability. Fasting RER values were also similar between HFs and HFpu mice, indicating that mice do not differ in the flexibility to switch to the mobilization and oxidation of stored lipids [68].

When mice were, however, challenged to switch from a fasting to a fed state, it became apparent that HFs mice made less efficient use of the carbohydrates in the diet. Also during the challenge with OxR, HFpu mice reached higher RER values than HFs mice, which indicates a higher level of glucose oxidation. The increase in RER during OxR was also observed in low-fat versus high-fat fed mice [9] and was accompanied by a reduction in oxygen consumption in low-fat versus high-fat fed mice. A metabolic switch from fat to glucose oxidation can lead to a small reduction in oxygen consumption [42], which is considered favourable during low-oxygen conditions. Mice that are less flexible, e.g. because of HF-feeding, proved to be less effective in this adaptation [9], which was also seen here in HFs versus HFpu mice. Both indirect calorimetry-based challenge tests, thus, show that HFs mice have a reduced capacity to increase glucose oxidation.

The mechanistic background behind the reduction in metabolic flexibility of HFs vs HFpu mice needs further investigation. The type of dietary fat does, unlike the amount of fat [69], not affect intestinal absorption of carbohydrates [70]; the differences in fatty acid composition, therefore, should not cause differences in the time course of fat absorption. HFs mice might, however, be less flexible because of a reduced mitochondrial capacity to oxidize carbohydrates. Chronic HF feeding is known to disturb β-oxidation, leading to increased accumulation of β-oxidative intermediates and reduced glucose oxidation: a condition that is referred to as ‘mitochondrial overload’ [71]. Saturated fatty acids are oxidized more slowly than unsaturated fatty acids [18, 72], but it is as yet unclear whether the changes in the rate of oxidation also increase incomplete FA oxidation. Alternatively, HFs mice might be more prone to use glucose for hepatic *de novo* lipogenesis (DNL) instead of using it for mitochondrial oxidation. Hepatic DNL is positively associated with obesity and other metabolic disorders [73], where it increases ectopic lipid storage [74]. Furthermore, increased hepatic DNL was, indeed, recently observed as a response to OxR in mice fed a high-fat versus low-fat diet [9].

### Conclusions

A diet high in SFAs versus a diet high in PUFAs, but similar n6/n3 ratio, did not affect many parameters of metabolic health in male mice, but led to an increase in mean adipocyte size in eWAT, ectopic lipid accumulation in liver and skeletal muscle, and to liver damage. Both indirect calorimetry-based challenge tests showed that HFs mice were less flexible in the switch from fat to carbohydrate use, which indicates a reduction in metabolic flexibility.

## Supporting Information

S1 Fig Hepatic lipid droplets after 5 days and 27 weeks of HF feeding. Representative images of the Oil-red-O stainings in liver after 5 days and 27 weeks of HFpu or HFs feeding. Pictures were used to visualise the extent of hepatic steatosis at the end of the study. The bar in each picture represents a distance of 100 μm.
**Methodology:** Liver tissue was immediately frozen after dissection, after which part of the left lobe was removed and sectioned at 7 μm with a cryostat (Leica Microsystems, Nussloch GmbH, Germany). Sections were made in four equally distant (distance: 56 μm) parts of the left lobe to achieve representative sections. Sections were, then, left at room temperature for 30 minutes and were fixed in 3.7% (v/v) buffered formalin and stained with Oil-red-O (Sigma-Aldrich, St Louis, MO, USA) as published [75] and with modifications as described [76]. Oil-red-O stainings were performed for 3 mice per group (randomly selected).
(TIF)

S2 Fig Triacylglycerol fatty acid composition in diets and liver. Shotgun lipidomics was used to determine partial composition of triacylglycerols in liver tissues and in the two diets. For triacylglycerol composition, the ratio of HFs/HFpu is plotted for total number of double bonds with dietary composition shown in white and liver composition in black (A). Differences in liver triacylglycerols indicate clear differences between HFs (blue, n = 6) and HFpu (yellow, n = 6) livers (B) focussing on the top 19 triacylglycerols based on abundance. Mean total number of carbon atoms in triacylglycerols is shown for liver tissue of HFs (blue) and HFpu (yellow) (C). Statistical analysis using Student’s *t*-test: * p<0.05, ** p<0.01, *** p<0.001.
(EPS)
