# Supplementary figures and images for: A Difference in Fatty Acid Composition of Isocaloric High-Fat Diets Alters Metabolic Flexibility in Male C57BL/6JOlaHsd Mice
Source: PLoS One. 2015 Jun 22;10(6):e0128515. doi: 10.1371/journal.pone.0128515 (PMC4476692; doi:10.1371/journal.pone.0128515)

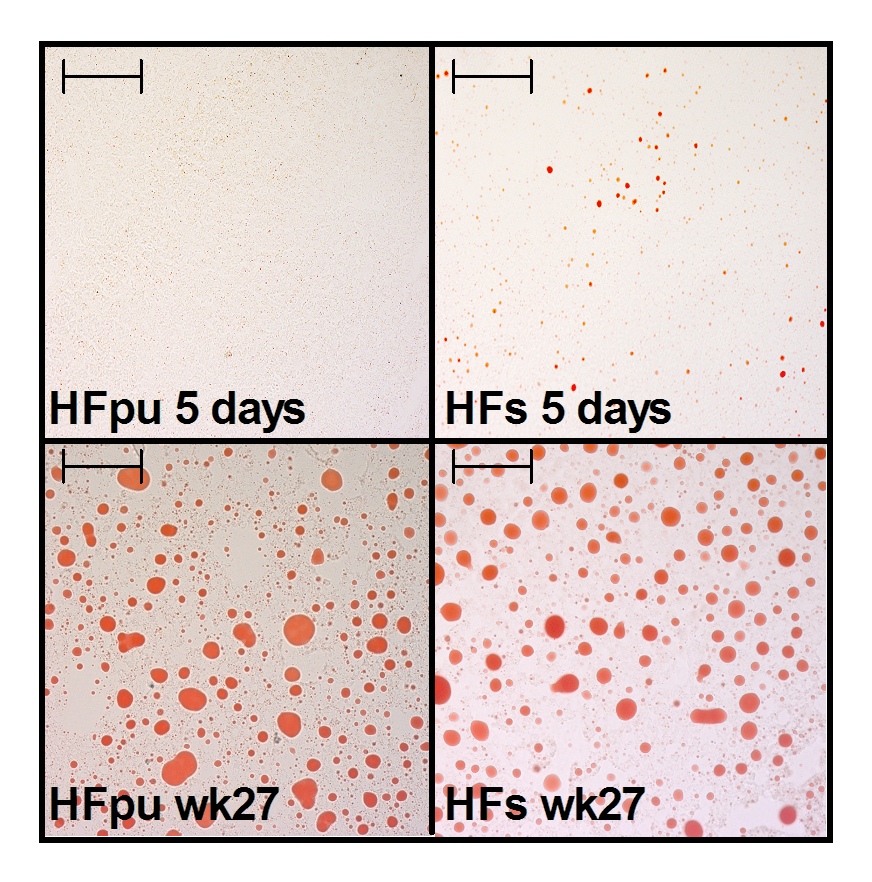

Supplement: S1 Fig — Representative images of the Oil-red-O stainings in liver after 5 days and 27 weeks of HFpu or HFs feeding. Pictures were used to visualise the extent of hepatic steatosis at the end of the study. The bar in each picture represents a distance of 100 μm. Methodology: Liver tissue was immediately frozen after dissection, after which part of the left lobe was removed and sectioned at 7 μm with a cryostat (Leica Microsystems, Nussloch GmbH, Germany). Sections were made in four equally distant (distance: 56 μm) parts of the left lobe to achieve representative sections. Sections were, then, left at room temperature for 30 minutes and were fixed in 3.7% (v/v) buffered formalin and stained with Oil-red-O (Sigma-Aldrich, St Louis, MO, USA) as published [75] and with modifications as described [76]. Oil-red-O stainings were performed for 3 mice per group (randomly selected). (TIF) [file pone.0128515.s001.tif]
